# Metformin regulates expression of DNA methyltransferases through the miR-148/-152 family in non-small lung cancer cells

**DOI:** 10.1186/s13148-023-01466-0

**Published:** 2023-03-23

**Authors:** Bo Bin Lee, Dongho Kim, Yujin Kim, Joungho Han, Young Mog Shim, Duk-Hwan Kim

**Affiliations:** 1 Department of Molecular Cell Biology, Samsung Biomedical Research Institute, Sungkyunkwan University School of Medicine, Suwon, 16419 Korea; 2 Yonsei New I1 Han Institute for Integrative Lung Cancer Research, Yonsei University College of Medicine, Seoul, 03772 Korea; 3 Department of Pathology, Samsung Medical Center, Sungkyunkwan University School of Medicine, Seoul, 06351 Korea; 4 Department of Thoracic and Cardiovascular Surgery, Samsung Medical Center, Sungkyunkwan University School of Medicine, Seoul, 06351 Korea; 5 Samsung Comprehensive Cancer CenterResearch Institute for Future Medicine S139-7, #50 Ilwon-dong, Gangnam-gu, Seoul, 06351 Korea

**Keywords:** Metformin, Lung cancer, microRNAs, DNMTs, Survival

## Abstract

**Background:**

To understand the molecular mechanisms involved in regulation of DNA methyltransferases (DNMTs) by metformin in non-small cell lung cancer (NSCLC) cells.

**Methods:**

Expression levels of DNMTs in response to metformin were analyzed in NSCLC cells. MicroRNAs regulating expression of DNMTs at the post-transcriptional level were searched using miRNA-target databases (miRDB and miRTarBase), TCGA RNASeqV2 lung cancer data, and miRNA-seq.

**Results:**

Metformin dose-dependently downregulated expression of DNMT1 and DNMT3a at the post-transcriptional level and expression of DNMT3b at the transcriptional level in A549 lung cancer cells. Activity of DNMTs was reduced by about 2.6-fold in A549 cells treated with 10 mM metformin for 72 h. miR-148/-152 family members (miR-148a, miR-148b, and miR-152) targeting the 3′UTR of DNMTs were associated with post-transcriptional regulation of DNMTs by metformin. Metformin upregulated expression of miR-148a, miR-148b, and miR-152 in A549 and H1650 cells. Transfection with an miR-148b plasmid or a mimic suppressed expression of DNMT1 and DNMT3b in A549 cells. Transfection with the miR-148a mimic in A549 and H1650 cells decreased the luciferase activity of DNMT1 3′UTR. A combination of metformin and cisplatin synergistically increased expression levels of miR-148/-152 family members but decreased expression of DNMTs in A549 cells. Low expression of miR-148b was associated with poor overall survival (HR = 2.56, 95% CI 1.09—6.47; *P* = 0.04) but not with recurrence-free survival.

**Conclusions:**

The present study suggests that metformin inhibits expression of DNMTs by upregulating miR-148/-152 family members in NSCLC cells.

**Supplementary Information:**

The online version contains supplementary material available at 10.1186/s13148-023-01466-0.

## Background

Lung cancer is the leading cause of cancer-related death in the world [1]. Despite significant advances in the diagnosis and treatment of this disease, its prognosis is extremely poor, with an overall five-year survival rate of approximately 20%. Such a poor prognosis is largely due to early micrometastatic spread of primary tumor cells into nearby lymph nodes or tissues through lymphatic and blood circulation. It is also partially due to early recurrence after curative resection. In addition, epigenetic modifications and molecular heterogeneity of a tumor pose significant challenges when treating lung cancer. Epigenetic alterations have been widely described in lung cancer and are potentially reversible. Accordingly, a number of epigenetic changes involved in lung cancer are potential targets for therapy.

DNA methylation is a key mechanism of epigenetic regulation, and dysregulated expression of DNA methyltransferases (DNMTs) has been reported in diverse types of human cancer. DNA methylation is established by interaction of DNMTs (DNMT1, DNMT3a, and DNMT3b) that can catalyze the transfer of a methyl group from the ubiquitous methyl group donor S-adenosyl-L-methionine (SAM) to the carbon 5 position of cytosine, which produces 5-methylcytosine. Overexpression of DNMTs and their relationships with DNA methylation in lung cancer have been reported by numerous groups. The mRNA levels of DNMT1 and DNMT3b are elevated in more than 50% of patients with non-small cell lung cancer (NSCLC) [2]. Altered expression levels of DNMT1, DNMT3a, and DNMT3b proteins have been found in patients with lung cancers, especially in smokers, and are correlated with hypermethylation of tumor suppressor genes [3]. In addition, the tobacco-specific carcinogen NNK prolongs the half-life of DNMT1 protein by activating AKT signaling associated with the ubiquitin-proteosome system and induces DNMT1 accumulation by inhibiting GSK3β–mediated DNMT1 degradation [4]. The average number of gene-specific hypermethylation events during lung carcinogenesis is correlated with increased protein levels of DNMT1 and DNMT3a [5]. DNMT3a is highly expressed in 58.5% (79 of 135) of lung adenocarcinomas. Such high expression is associated with the histologically lepidic subtype [6]. Based on these reports, inhibition of DNMTs could be a promising approach for lung cancer treatment. However, there are currently no available DNMTs inhibitors for lung cancer.

Metformin is an oral antidiabetic drug used to treat type II diabetes. It has also been tested as an anticancer agent because of its ability to suppress tumor growth and increase cell death. Increasing reports have shown the effect of metformin on epigenetic regulation in lung cancer. For example, metformin can inhibit the proliferation of lung cancer cells by reducing miR-222, which targets p27, p57, and PTEN [7]. Metformin suppresses the growth, migration, and invasion of A549 cells by increasing the expression of miR-7, which downregulates p-AKT and p-mTOR expression through AMPK [8]. Metformin can also decrease the growth and invasion of lung cancer cells by up-regulating miR-381, which functions as a tumor suppressor [9]. Tissue-specific indirect and direct effects of metformin on histone modification have also been shown in various types of cancer. Metformin reduces histone H3 lysine 27 trimethylation (H3K27me3) and polycomb repressor complex 2 (PRC2) in ovarian cancer cells [10]. Metformin hinders metastasis of prostate cancer cells by stimulating the expression of histone methyltransferase SETD2 (SET Domain Containing 2) while decreasing EZH2 (Enhancer of Zeste Homolog 2) and H3K27me3 levels [11]. We have also reported that metformin can reduce H3K4me3 level at promoters of positive cell cycle regulatory genes such as E2F8 by downregulating H3K4 methyltransferase MLL2 in lung cancer cells [12]

Despite growing evidence showing that metformin can regulate miRNA and histone modification, the effect of metformin on expression of DNMTs in lung cancer and the underlying molecular mechanisms have not been elucidated. In the present study, we demonstrated that metformin suppressed expression of DNMTs by upregulating miR-148/-152 family members in NSCLC cells.

## Methods

### Cell culture

Lung cancer cell lines (A549 and H1650) were purchased from the American Type Culture Collection (Rockville, MD). These cells have been authenticated using Short Tandem Repeat (STR) profiling at Samsung Medical Center (Seoul, Korea) within the last five years. A549 and H1650 cells were cultured in RPMI 1640 medium containing 10 mM HEPES and complete RPMI 1640 medium, respectively, supplemented with 10% heat-inactivated fetal bovine serum (FBS) (GIBCO-BRL, Invitrogen, Carlsbad, CA) in a humidified incubator at 37 °C with 5% CO_2_. Metformin (Sigma-Aldrich, St. Louis, MO) was dissolved in water and then diluted with phosphate-buffered saline (pH 7.4). All experiments were performed with mycoplasma-free cells.

### Quantitative real-time RT-PCR

Total RNAs were extracted from A549 and H1650 cells using a Qiagen RNeasy Mini Kit (Valencia, CA). cDNA was synthesized using a QuantiTect Reverse Transcription Kit (Qiagen) for DNMTs or miRNA First Strand cDNA Synthesis Kit (Agilent, Santa Clara, CA) with tailing reaction for miRNAs. Quantitative real-time PCR was performed with an ABI PRISM 7900HT Sequence Detection system (Applied Biosystems, Foster City, CA) using SYBR Green PCR Master Mix (Applied Biosystems) according to the manufacturer’s protocol. Relative RNA expression level was calculated by the 2^−ΔΔCT^ method, and GAPDH and U6 were used as internal controls for mRNA and miRNA, respectively. Primer sequences are listed in Additional file 1.

### Immunoblot analysis

Cultured cells were collected, rinsed with chilled PBS, and lysed in buffer containing 2.5 mM sodium pyrophosphate, 1 mM beta-glycerophosphate, 150 mM NaCl, 1% sodium deoxycholate, 20 mM Tris–HCl (pH 7.5), 1 mM EGTA, 1% NP-40, 1 mM Na_3_VO_4_, 1 mM Na_2_EDTA, and 1 µg/ml leupeptin. The lysis buffer was supplemented with 1 mM PMSF immediately before cell lysis. Lysates were sonicated and then centrifuged at 13,000 rpm for 15 min at 4 °C to pellet insoluble proteins. Cell lysates were subjected to 8% or 12% sodium dodecyl sulphate–polyacrylamide gel electrophoresis (SDS-PAGE) and then transferred to polyvinylidene difluoride (PVDF) membranes (Millipore, Bedford, MA). Nonspecific binding sites were blocked using 5% non-fat dry milk (Bio-Rad, Hercules, CA) in Tris-buffered saline containing 0.1% Tween 20 (TBS-T) for 1 h at room temperature. After blocking, membranes were incubated with primary antibodies specific for DNMT1, DNMT3a, and DNMT3b (Cell Signaling Technology 5425, Danvers, MA) at 4 °C overnight. These membranes were then washed with TBS-T three times (5 min each) and incubated with horseradish peroxidase (HRP)‐conjugated secondary antibody for 1 h at room temperature. Protein bands were visualized using a highly sensitive SuperSignal West Pico Chemiluminescent Substrate (Invitrogen) for detecting HRP.

### DNMT activity assay

Nuclear extracts were prepared from A549 cells treated with metformin using an EpiQuik™ Nuclear Extraction Kit (Epigentek, Farmingdale, NY) according to the manufacturer's protocol. Protein concentrations of nuclear extracts were determined with a Pierce™ Bioinchoninic Acid Assay (BCA) kit (Thermo Fisher Scientific, Waltham, MA). DNA methyltransferase (DNMT) activity (de novo, maintenance) was measured using 4–20 µg of protein and an EpiQuik DNA Methyltransferase Activity/Inhibition Assay Kit (Epigentek) in accordance with the user manual. Results were expressed as absorbance units detected by microplate reader at 450 nm, and DNMT enzymatic activity was calculated with the following formula: DNMT activity (OD/h/mg) = (sample OD − blank OD) × 1,000/[(protein amount (µg) × incubation time (hours)].

### Screening of miRNAs targeting DNMTs

Two online databases, miRDB (http://www.mirdb.org) and miRTarBase (https://bio.tools/mirtarbase), were used to predict miRNAs with potential to regulate DNMT genes at the post-transcriptional level. According to the guidelines of each database, miRNAs with a target score of 80 or higher that could simultaneously target two or more of the DNMT1, DNMT3a, and DNMT3b genes were selected.

### Analysis of TCGA RNASeqV2 data

The Cancer Genome Atlas (TCGA) RNASeqV2 lung cancer data were used to confirm expression status of the miR-148/-152 family with a high target score among miRNAs selected from prediction databases. Raw counts of miRNAs were extracted from 45 lung cancer patients with adjacent normal tissue samples out of 565 LUAD (lung adenocarcinoma) patients with miRNA expression data (level 3). Raw counts were finally extracted from 18 patients with matching RNASeqV2 data for tumor and matched normal tissues.

### MicroRNA-seq analysis

A549 and H1650 cells were treated with 10 mM metformin for 72 h. After the treatment, miRNAs were isolated from these cells using an miRNeasy Mini Kit (Qiagen, cat No./ID: 217,004) following the manufacturer's protocol. The quality of extracted miRNAs was first assessed using a NanoDrop 2000 spectrophotometer (Thermo Fisher Scientific). RNA integrity in samples with total RNA greater than 2 μg, an optical density (OD) of 260/280 ≥ 2.0, and OD 260/230 ≥ 2.0 was further analyzed using a 2100 Bioanalyzer (Agilent). Samples with RIN (RNA integrity number) ≥ 7 and ribosomal RNA (rRNA) 28S/18S ≥ 0.7 were selected. Small RNAs (18–30-nucleotide segments) were separated from total RNA by PAGE on a Bioanalyzer. An RNA library was prepared according to the protocol of a NEBNext Multiplex Small RNA Library Prep Kit for Illumina (Index Primers 1–48) and sequenced using an Illumina NextSeq500 platform according to the vendor’s recommended protocol. After sequencing, the quality of produced data was determined by Phred quality score, and reads with an average score over 20 were accepted as good quality. Read counts for each miRNA were normalized using quantile normalization between samples. Afterward, through DEG analysis, miRNAs whose expression levels were increased or decreased by more than two-fold with a normalized RC(log) value of 3 or more and a *p*-value of 0.05 or less as a result of t-test were selected as differentially expressed miRNAs. Using MeV software, hierarchical clustering analysis was performed, and genes whose expression levels were increased more than 1.5-fold by metformin were further classified through gene ontology analysis. To understand functional roles of dysregulated miRNAs, DAVID bioinformatics resources (v6.8; https://david.ncifcrf.gov/) were used for gene ontology (GO) analysis, and DIANA-miRPath v3.0 (http://www.microrna.gr/miRPathv3) was employed for Kyoto Encyclopedia of Genes and Genomes (KEGG) pathway analysis.

### Transfection assay

To demonstrate whether miR-148/-152 family members could regulate expression of DNMTs directly, we constructed an expression clone of miR-148b precursor in the pEZX-MR04 vector. A mimic of miR-148b that exhibited the same activity as endogenous miRNA and a single-stranded synthetic inhibitor having a complementary sequence to inhibit this activity were purchased from Bioneer Co. (Daejeon, South Korea). For transient transfection, A549 cells were plated onto 12-well plates. Cells were transfected at approximately 70–80% confluency with miR-148b-3p construct, miR-148b mimic, or miR-148b inhibitor using Lipofectamine RNAiMAX reagent (Invitrogen, Cat. No. 13778150), following the manufacturer’s protocol. At 24 h post-transfection, some cells were treated with 10 mM metformin for an additional 72 h. Expression levels of DNMTs were then measured using western blot.

### Luciferase reporter assay

Putative interactions between miR-148a-3p and DNMT1 3'UTR were analyzed through a luciferase reporter assay. miRNA 3′UTR target expression clone for Human DNMT1 (NM_001130823.2) was purchased from GeneCopoeia (Rockville, MD, Cat no. HmiT094835-MT06). For DNMT1 3′UTR reporter assay, Luc-DNMT1-3′UTR constructs (DNMT1-WT-3′UTR and DNMT1-MT-3′UTR) containing a potential wild-type or a mutant binding sequence were created in the pEZX-MT06 reporter vector (GeneCopoeia, Cat no. CmiT000001-MT06). The DNMT1-WT-3′UTR or DNMT1-MT-3′UTR plasmid was transfected into A549 or H1650 cells alone or in combination with a miR-148a mimic or an miR-148a inhibitor in a 96-well plate using Lipofectamine RNAiMAX reagent (Invitrogen, Cat. No. 13778150) in 5.0 μl Opti-MEM reduced serum medium (Thermo Fisher). Some cells were treated with 10 mM metformin at 24 h post-transfection. Cells were harvested at 24 h after transfection or 72 h after metformin treatment, and luciferase activity was measured using a Gaussia luciferase assay kit (Promega, Madison, WI) according to the manufacturer's instructions. Independent experiments were performed in triplicate for each plasmid construct.

### Coefficient of drug interaction (CDI)

To understand the combined effect of metformin and cisplatin on expression of miR-148/-152 and cisplatin in lung cancer cells, we first calculated the coefficient of drug interaction (CDI) to analyze the nature of drug interactions. A549 cells were treated with both metformin (0 mM and 5 mM) and cisplatin (0 mM, 6.25 mM, 12.5 mM, 25 mM, and 50 mM) for 72 h. CDI was calculated as follows: CDI = AB/(A × B), where AB was the OD ratio of the combination treatment groups to the control group, and A or B was the OD ratio of the single treatment group to the control group.

### Statistical analysis

For univariate analysis, *t*-test or analysis of variance (ANOVA) was used for the continuous variables such as miR-148/-152 expression levels. The means between an experimental group and a control group were compared using the Student t-test. Correlation of the two continuous variables was assessed using Pearson's correlation coefficient. The effect of miR-148/-152 expression on overall or recurrence-free survival of NSCLC patients was estimated using the Kaplan–Meier survival curve, and the difference between survival curves of the two groups was evaluated by log-rank test. The hazard ratio of low miR-148/-152 family expression for survival was computed using Cox proportional hazards analysis after adjusting for potential confounding factors. All statistical analyses were conducted using R software (version 4.2.1).

## Results

### Metformin regulates expression of DNMT1 and DNMT3a at the post-transcriptional level

**Fig. 1:**
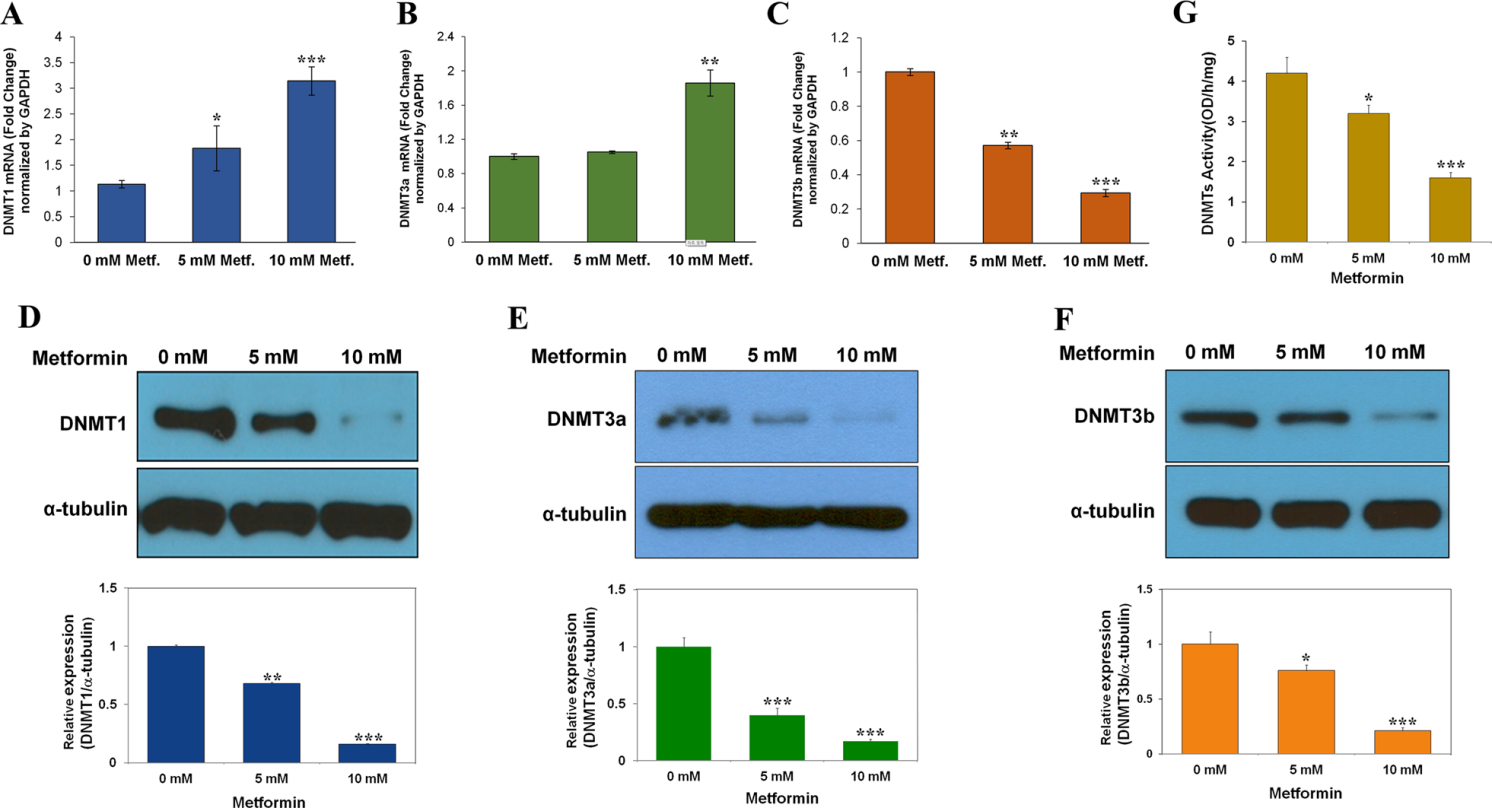
Metformin suppresses protein expression and activity of DNMTs. A549 cells were treated with 0 mM, 5 mM, or 10 mM metformin for 72 h. **A**–**C** mRNA levels of DNMTs were measured by qRT‐PCR. **D**–**F** Protein levels of DNMTs were determined using western blot with α-tubulin as an internal loading control. **G** Nuclear proteins were extracted using an EpiQuik™ Nuclear Extraction Kit, and the activity level (OD/h/mg nuclear protein) of total DNMTs was measured using an EpiQuik™ DNA Methyltransferase Activity/Inhibition Assay Kit. Results are shown as mean ± standard deviation (n = 3). *P < 0.05, **P < 0.01, ***P < 0.001

To understand the effect of metformin on expression of DNMTs (DNMT1, DNMT3a, and DNMT3b), A549 NSCLC cells were treated with 0 mM, 5 mM, and 10 mM of metformin for 72 h. DNMT1 and DNMT3a mRNA levels increased in a dose-dependent manner (Fig. 1A, B), whereas DNMT3b mRNA levels decreased with increasing dosage of metformin (Fig. 1C). Unlike mRNA changes of DNMT1 or DNMT3a, protein levels of the three DNMTs were decreased in response to metformin (Fig. 1D–F). Protein levels of DNMT1, DNMT3a, and DNMT3b decreased by 8.5-, 7.7-, and 6.4-fold in response to metformin treatment, respectively. Activity of DNMTs also decreased by about 2.6-fold in cells treated with 10 mM metformin (Fig. 1G). These results suggest that metformin regulates the expression of DNMTs in NSCLC cells at the transcriptional or post-transcriptional level.

### The miR-148/-152 family is a potential target of metformin in NSCLC cells

**Fig. 2:**
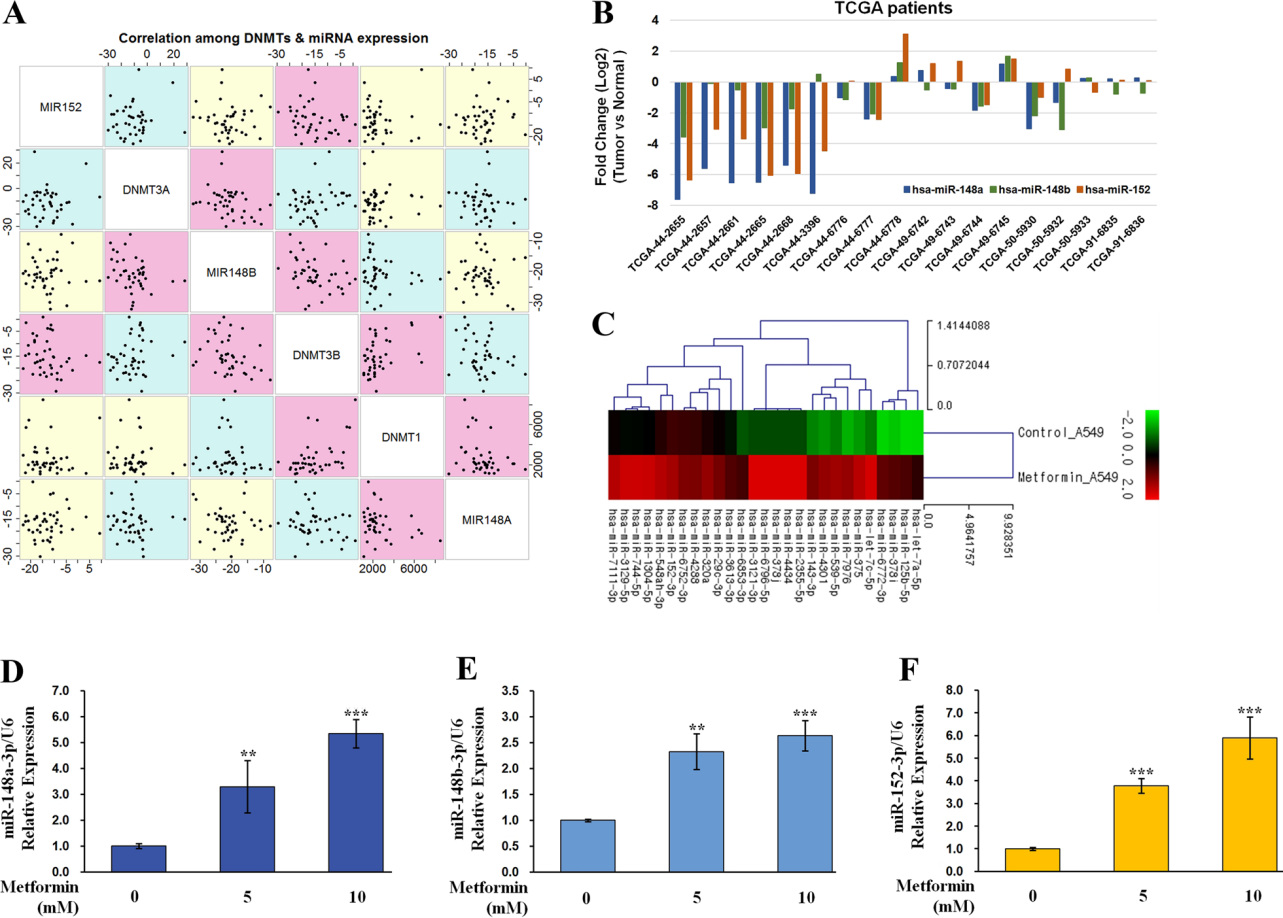
miR-148/-152 family members target DNMTs 3′UTR. **A** Correlations among miR-148/-152 family members and DNMT expression levels in tumor tissues from 42 NSCLC cancer patients were analyzed using Spearman’s correlation coefficient. Magenta color indicates *P* < 0.05. **B** Expression patterns of the miR-148/-152 family were analyzed in tumor tissues relative to normal tissues of 18 lung adenocarcinoma patients selected from the TCGA dataset. **C** Hierarchical clustering analysis of differentially expressed miRNAs between A549 cells treated with metformin or not. Red and green represent upregulated and downregulated miRNAs, respectively. **D–F** A549 cells were treated with 0 mM and 10 mM metformin for 72 h. Expression levels of miR-148/-152 family members were measured by qRT‐PCR. Expression levels of miR-148a-3p (**D**), miR-148b-3p (**E**), and miR-152-3p (**F**) were normalized to that of U6 small nuclear RNA. Data shown are mean ± standard deviation (n = 3), and significant differences are indicated by *P < 0.05, **P < 0.01, ***P < 0.001

To identify miRNAs with the potential to regulate expression of DNMTs at the post-transcriptional level, we first searched the 3′UTR of DNMTs using two common bioinformatic algorithms, miRDB and miRTarBase. Based on guidelines of each algorithm, we selected miRNAs from two groups among those with a target score of 80 or higher: 1) miRNAs (miR-148, miR-29 and miR-548) that could simultaneously target two or more of the DNMT1, DNMT3a, and DNMT3b genes and 2) miRNAs (miR-200, miR-101, miR-2276, miR-4708 and miR-7152) that could target each of the three DNMTs. Second, a relationship between candidate miRNAs and DNMT expression was analyzed using HT-12 array data of 42 NSCLCs reported previously [12]. No relationship was found between miR-152 expression and expression of the three DNMTs. However, miR-148a expression was inversely correlated with the expression of DNMT1 (ρ = − 0.31, *P* = 0.04), whereas miR-148b expression showed negative correlation with the expression of DNMT3A (ρ = − 0.38, *P* = 0.02) and DNMT3B (ρ = − 0.33, *P* = 0.03) in tumor tissues from 42 NSCLC patients (Fig. 2A).

Third, to determine whether the selected miR-148/-152 family members were significantly reduced in lung cancer patients of other races, we analyzed TCGA RNASeqV2 data. Raw counts of miRNAs were extracted from 45 lung cancer patients with adjacent normal tissue samples out of 565 LUAD patients with miRNA expression data (level 3). Raw counts were finally extracted from 18 patients with matching RNASeqV2 data. Using the LIMMA package of R language, we found that the expression of miR-148/-152 family members was significantly reduced in 12 of these 18 patients (Fig. 2B). Fourth, miRNAs regulated by metformin were investigated using miRNA-seq. Through DEG analysis, miRNAs whose expression levels were increased or decreased by more than two-fold with a normalized RC(log) value of 3 or more and a *p*-value of 0.05 or less were selected. Hierarchical clustering (Fig. 2C; Additional file 2) and gene ontology analysis (Additional file 3) were performed using MeV program. DNMT1 was found to be related to miR-152 in A549 cells (Fig. 2C) and miR-148b in H1650 cells (Additional file 2). For validation of miR-148/-152 selection, qRT-PCR was performed for A549 and H1650 cells treated with metformin for 72 h. We found that expression levels of the miR-148/-152 family members were increased depending on metformin concentration (Fig. 2D–F). Considering these observations, we selected the miR-148/-152 family as a potential target since its expression was increased in response to metformin and correlated with expression of DNMTs in lung cancer cells.

### miR-148b directly controls DNMT1 and DNMT3b in lung cancer cells.

**Fig. 3:**
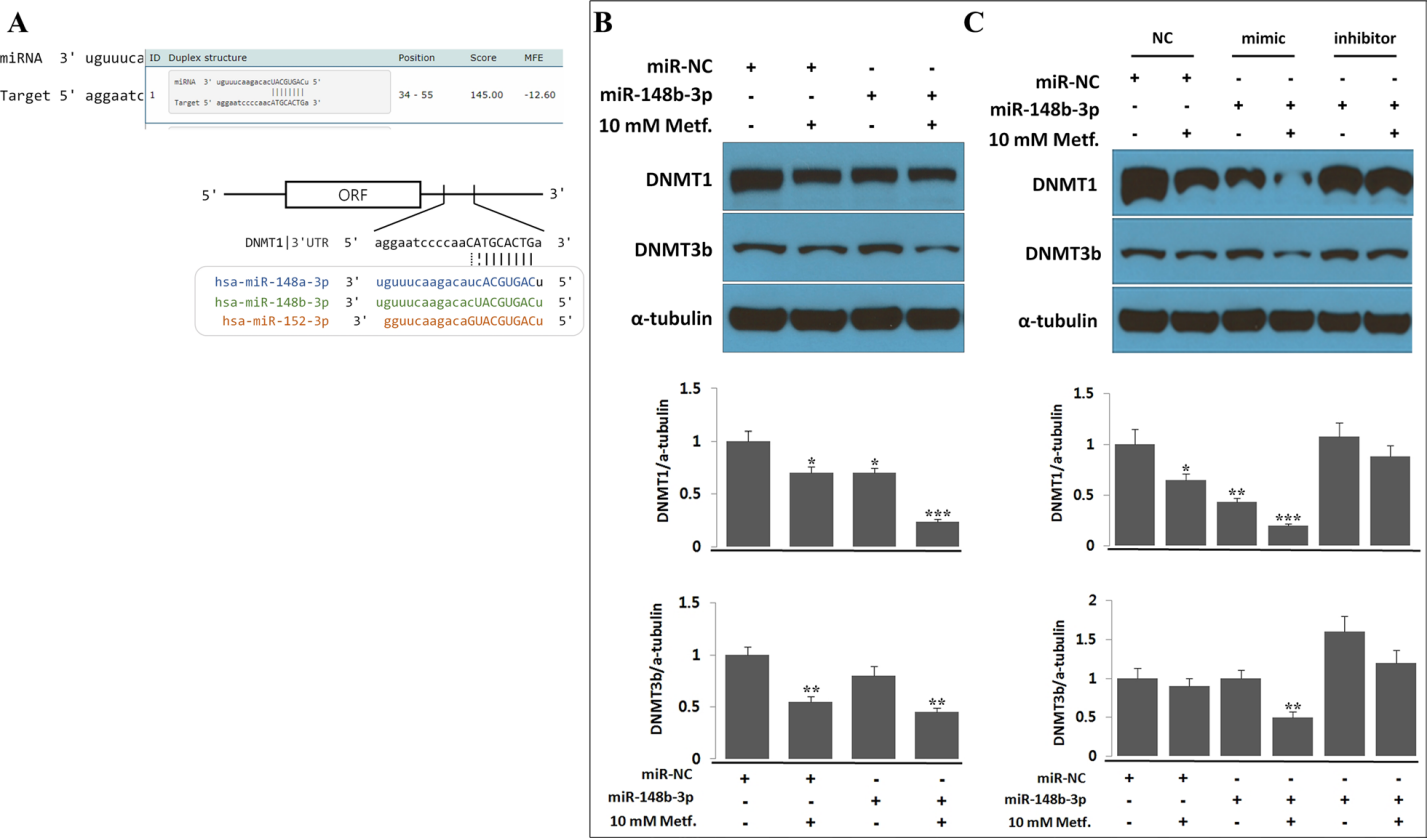
miR-148b negatively regulates DNMT1 and DNMT3b expression in A549 cells. **A** Sequences of miR-148/-152 precursor family members targeting DNMT1 3′UTR. **B**, **C** miR-148b-3p precursor was cloned into a pEZX-MT04 vector. miR-148b-3p plasmid (**B**) or miR-148b-3p mimic or inhibitor (**C**) was transfected into A549 cells using Lipofectamine® RNAiMAX. Cells were cultured for 24 h after transfection and then treated with 10 mM metformin for an additional 72 h. After 72 h, protein levels of DNMT1 and DNMT3b were determined using western blot, and α-tubulin was used as an internal loading control. NC, scrambled oligonucleotides as a negative control. Data are presented as mean ± standard deviation (n = 3). Statistical analysis: *P < 0.05, **P < 0.05, ***P < 0.001

We cloned a precursor miR-148b nucleotide (Fig. 3A) into the pEZX-MR04 vector to study whether miR-148b could modulate the expression of DNMTs under the influence of metformin. When the miR-148b clone was transfected into A549 cells, protein levels of DNMT1 and DNMT3b were reduced by at least 30% compared to those in the control. They were suppressed by more than 50% when cells were cultured with 10 mM metformin for an additional 72 h following a 24 h incubation after transfection. (Fig. 3B). When the miR-148b mimic was transfected, expression levels of DNMT1 and DNMT3b were reduced compared to those in cells treated with a negative control. When cells were treated with metformin, their levels were reduced by greater than 50% (Fig. 3C). However, in the presence of an miR-148-3p inhibitor, the effect of metformin on DNMT expression was minimal (Fig. 3C). Cell death had an inverse relationship with the expression level of miR-148b (Additional file 4).

### miR-148a controls luciferase activity of DNMT1 3′UTR

**Fig. 4:**
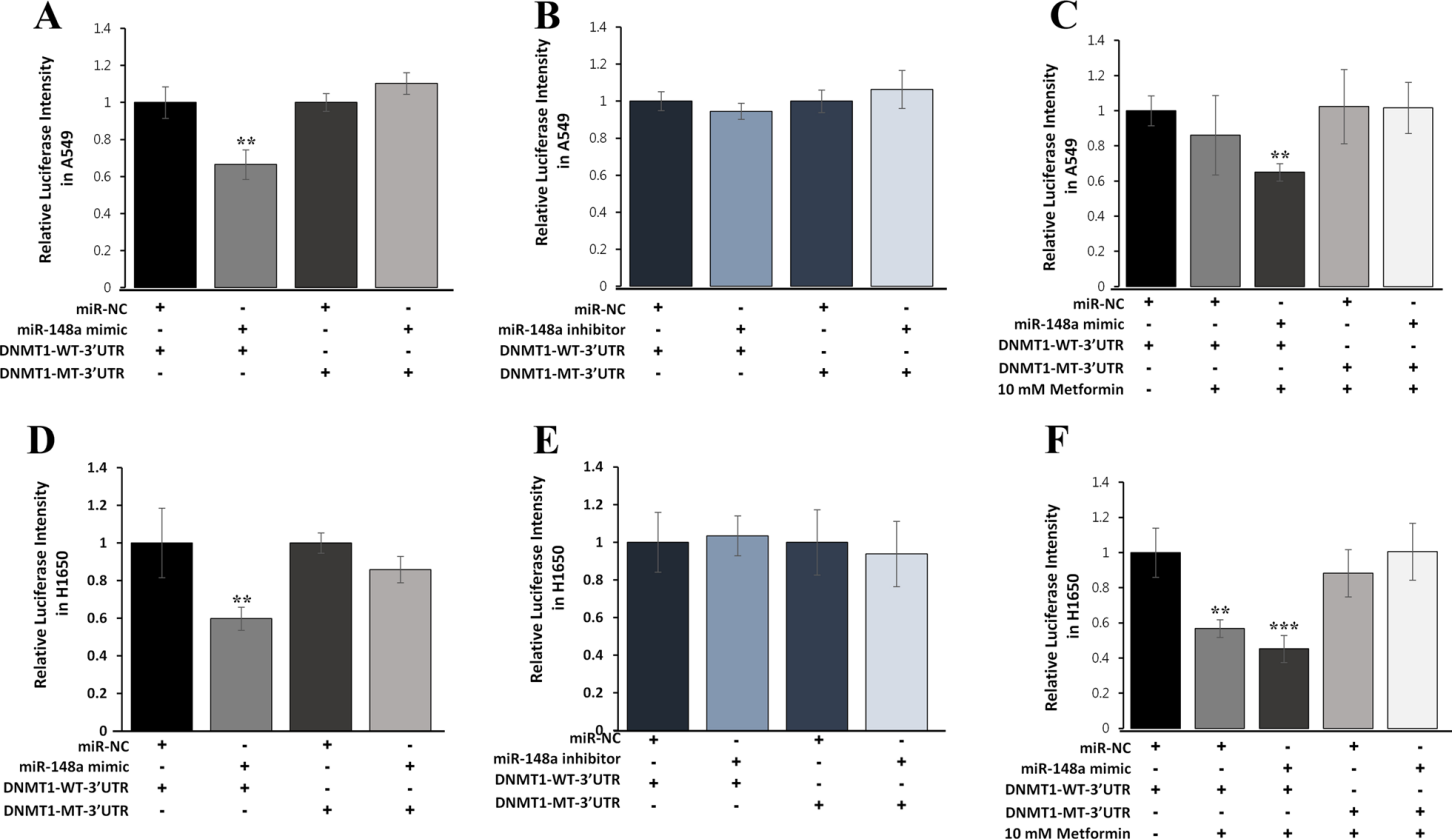
miR-148a suppresses luciferase reporter activity of DNMT1 3′UTR in NSCLC cells. Luciferase reporter assay was performed in A549 (**A**–**C**) and H1650 cells (**D**–**F**) co-transfected with an miR-148a mimic or an miR-148a inhibitor at a final concentration of 50 nM in combination with DNMT1-WT/MT-3′UTR reporter in a 96-well plate using Lipofectamine® RNAiMAX following the manufacturer's instructions. Some cells were incubated for 24 h after transfection, and others were subsequently treated with 10 mM metformin for 72 h. Luciferase activities were measured using a Gaussia luciferase assay kit (Promega) according to the manufacturer's protocol. Data represent mean ± standard deviation (SD) from three independent experiments. *P < 0.05, **P < 0.05, ***P < 0.001

Luciferase assay was performed using A549 cells (Fig. 4A–C) and H1650 cells (Fig. 4D–F) to understand whether miR-148/-152 family members could interact with DNMT1 3′UTR. A Luc-DNMT1-3′UTR construct containing a potential binding sequence (WT48-54 DNMT1 3′UTR) and a construct with a mutant binding sequence (MT48-54 DNMT1 3′UTR) were created in the pEZX-MT06 reporter vector. Transfection of the miR-148a mimic in A549 and H1650 cells inhibited the activity of the luciferase reporter vector containing the WT48-54 DNMT1 3′UTR by 40%. However, it did not affect the luciferase activity of the MT48-54 DNMT1 3′UTR (Fig. 4A, D). Inhibition of miR-148a had no effect on the luciferase activity of DNMT1 3′UTR (Fig. 4B, E). In addition, luciferase activity was further inhibited when both cell lines were additionally treated with metformin (Fig. 4C, F). These observations suggest that DNMT1 is a potential target for miR-148a and that its 3′UTR is a functional target site for miR-148a in NSCLC cells.

### Effect of metformin combined with cisplatin on miR-148/-152 family members and DNMTs in NSCLC cells

**Fig. 5:**
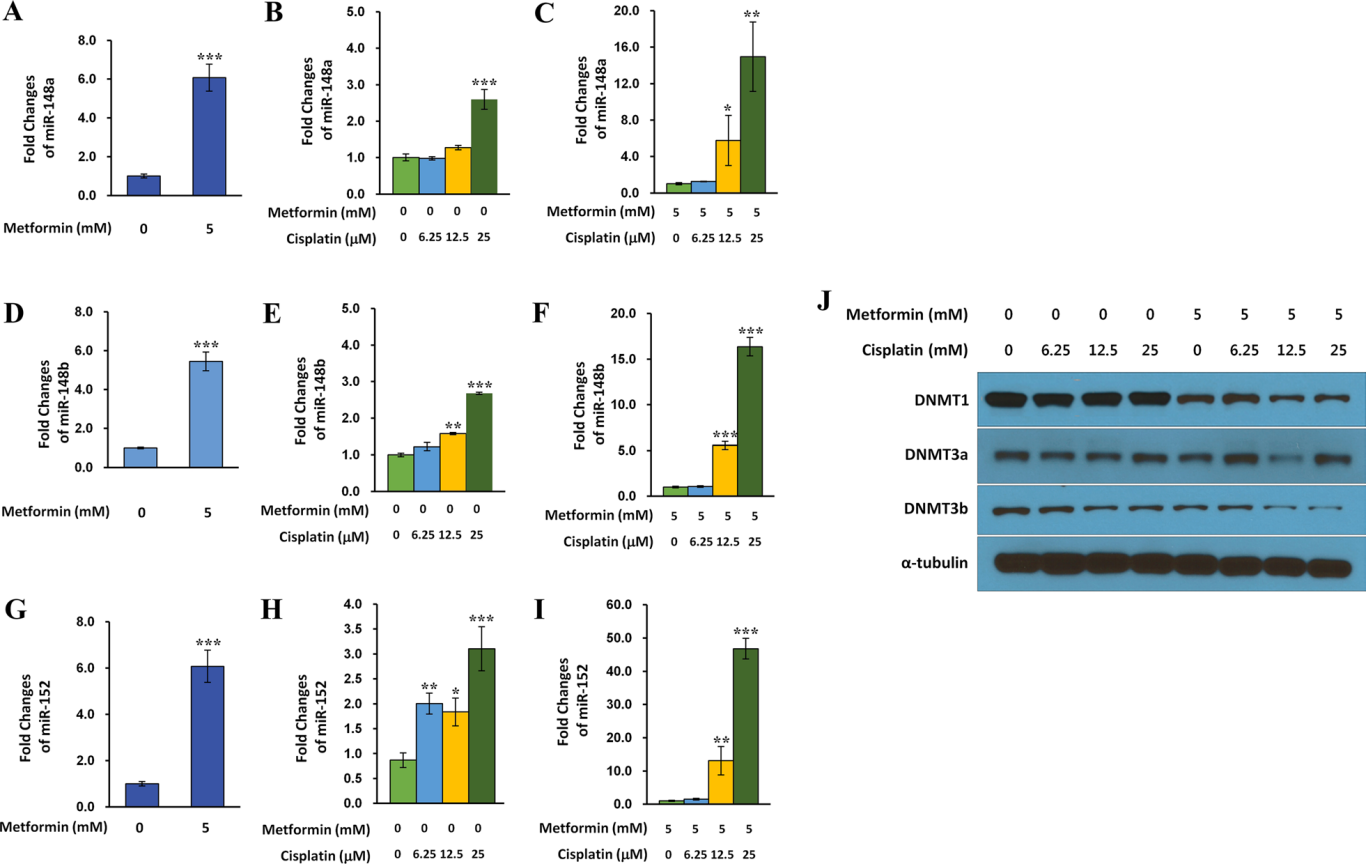
Metformin and cisplatin affect expression of miR-148/-152 and DNMTs synergistically. Effects of metformin combined with cisplatin on the expression of miR-148/-152 family members and DNMTs were analyzed in A549 cells. Metformin (0 mM and 5 mM) and cisplatin (0 mM, 6.25 mM, 12.5 mM, and 25 mM) were used alone or in combination. After 72 h of drug treatment, expression levels of miR-148-/-152 family members (**A**–**I**) and DNMTs (**J**) were analyzed using qRT-PCR and western blot, respectively. The graph shows mean ± standard deviation of relative expression of miR-148/-152 family members from three independent experiments. Fold change (y-axis) represents expression of the target mRNAs at other concentrations of metformin and cisplatin relative to expression after treatment with 0 mM of metformin (**A**, **D**, **G**), 0 mM of metformin and cisplatin (**B**, **E**, **H**), or 5 mM of metformin and 0 mM of cisplatin. Data are expressed as mean ± standard deviation (n = 3). The means between independent two groups are compared by Student t-test (*P < 0.05, **P < 0.01, ***P < 0.001)

To investigate the effect of metformin combined with cisplatin on the expression of miR-148/-152 family members and DNMTs, drug interaction was analyzed by measuring the CDI between cisplatin and metformin. After A549 cells were treated with various concentrations of the two drugs, the CDI was measured. Within a wide concentration range, CDI was less than 0.7, showing a meaningful synergistic effect (Additional file 5). Quantitative RT-PCR of miR-148/-152 family members was performed to investigate whether metformin and cisplatin had a synergistic effect on the expression of miR-148/-152 family members. Like metformin (Fig. 5A, D, G), cisplatin also led to a maximum three-fold increase in the expression of miR-148/-152 family members in a concentration-dependent manner, although the fold change was small in comparison with metformin (Fig. 5B, E, H). Expression levels of miR-148/-152 family members were increased synergistically when cells were simultaneously treated with metformin and cisplatin rather than treated with them individually (Fig. 5C, F, I). In contrast, the expression of DNMT1 and DNMT3b was decreased synergistically after treatment with combined metformin and cisplatin. DNMT3a also showed a slightly decreasing tendency (Fig. 5J).

### Clinicopathological characteristics of miR-148/-152 family members

**Fig. 6:**
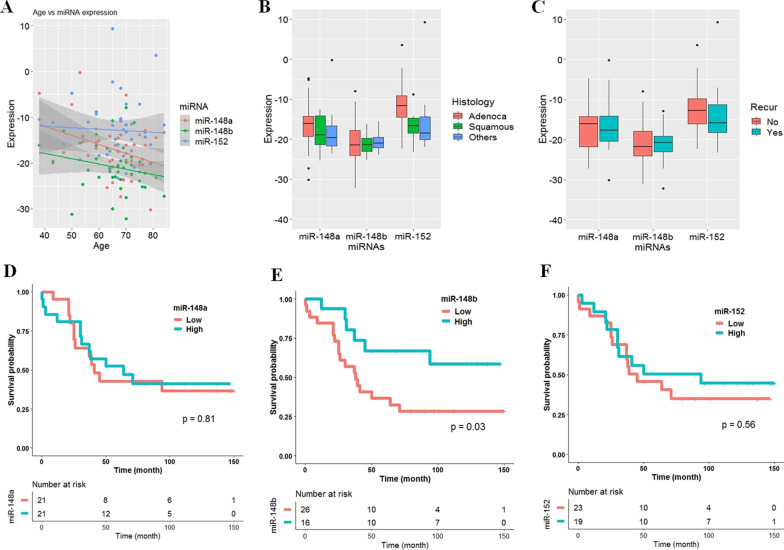
Negative effect of low miR-148b expression on overall survival of 42 NSCLC patients. **A**–**C** Expression levels of miR-148a in tumor tissues from 42 NSCLC patients were compared according to age (**A**), histology (**B**), or recurrence (**C**). **D**–**F** Relationships between overall survival and expressions of miR-148a (**D**), miR-148b (**E**), and miR-152 (**F**) in 42 NSCLC patients were analyzed using Kaplan–Meier survival estimates. *P*-value was calculated using the log-rank test

Clinicopathological significance of miR-148/-152 family members was analyzed in tumor tissues from 42 NSCLC patients (Additional file 6). No association was found between the expression of miR-148/-152 family members and histology, recurrence (Fig. 6B, C), sex, pathologic stage, smoking status, lymphatic invasion, lymph node metastasis, venous invasion, or nervous invasion. In addition, tumor size was not correlated with expression of miR-148/-152 family members (Additional file 7), but miR-148a expression was inversely correlated with patient age (- 0.33, *P* = 0.03; Fig. 6A; Additional file 7). To analyze the effect of expression of miR-148/-152 family members on patient survival, patients were divided into high- and low-expression groups by the mean value of miR-148/-152 family members. The median follow-up period of patients was 5.3 years. The expression of miR-148a and miR-152 did not show a relationship with patient survival (Fig. 6D–F; Additional file 8). However, patients with low expression of miR-148b showed significantly poor overall survival (*P* = 0.03, Fig. 6D). Cox proportional hazards analysis showed that overall survival of patients with low miR-148b expression was 2.56 times (95% CI 1.09—6.47; *P* = 0.04) poorer than that of those with high expression of miR-148b after adjusting for confounding factors (Additional file 9). Five-year overall survival rates of patients with low and high miR-148b expression were 37% and 68%, respectively.

## Discussion

In this study, we investigated the potential of metformin as an inhibitor of DNMTs and the underlying mechanisms of DNMT regulation in NSCLC cells. A couple of groups have reported that metformin can downregulate DNMT expression in human cancer cells. The combination of metformin and berberine synergistically inhibited DNMT1 expression by reducing transcription factor SP1 and 3‐phosphoinositide‐dependent protein kinase‐1 (PDPK1) expression in NSCLC cells [13]. Metformin-activated AMPK increased DNMT3A activity in liver and breast cancer cells [14]. The present study showed that metformin decreased DNMT expression at the transcriptional or post-transcriptional level through upregulation of miR-148/-152 family members in NSCLC cells (Additional file 10), suggesting that metformin has potential as a DNMT inhibitor.

The miR-148/-152 family consists of miR-148a, miR-148b, and miR-152, which are located at human chromosomes 7, 12, and 17, respectively. miR-148/-152 family members are known to act as tumor suppressors in NSCLC, playing important roles in initiation, growth, and metastasis through various regulatory mechanisms [15]. Mounting evidence has indicated that miR-148/-152 family members target the DNMT1 3′UTR in the development and progression of human cancer. DNMT1 was regulated by interleukin-6-dependent miR-148a, miR-152, and miR-301 in human malignant cholangiocytes [16]. Ectopic overexpression of miR-148a decreased DNMT1 expression in acute myeloid leukemia [17], gastric cancer [18], hepatocellular carcinoma [19], pancreatic cancer [20], and prostate cancer cells [21]. miR-148a also repressed proliferation of bladder cancer cells by establishing a positive feedback loop between ERBB3/AKT2/c-myc signaling and DNMT1 [22] or by regulating expression levels of DNMT1 and undifferentiated embryonic cell transcription factor 1 (UTF1) in cervical cancer cells [23]. Transfection of miR-148b or miR-152 in pancreatic cancer cells reactivated tumor suppressor genes such as BNIP3, SPARC, PENK, and TFPI-2 by downregulating DNMT1 [24]. Upregulation of miR-148b reversed cisplatin resistance in NSCLC cells by targeting the DNMT1 3′UTR, but not DNMT3a or DNMT3b [25]. miRNA-148b suppressed cell cycle progression and facilitated cell apoptosis by regulating DNMT1 in cervical cancer [26] and endometrial cancer [27] in a caspase-3 dependent manner. miR-152 inhibited the proliferation, migration, or invasion by targeting the DNMT1 3′UTR directly in hepatitis B virus-related hepatocellular carcinoma [28], ovarian cancer [29], glioma [30], nasopharyngeal cancer [31] and bladder cancer cells [32]. All these findings indicate that DNMTs are downstream targets of miR-148/-152 family members in various types of cancer.

The miR-148/-152 family members have been reported to induce sensitization to cisplatin chemotherapy by targeting DNMT1. Sui et al. [25] reported that miR-148b is substantially downregulated in cisplatin-resistant NSCLC cells, and that upregulation of miR-148b can increase the sensitivity to cisplatin by negatively regulating DNMT1 expression. Overexpression of miR-152 upregulated cisplatin sensitivity both in vitro and in vivo by suppressing DNMT1 directly in ovarian cancer cells [29]. In this study, the effect of cisplatin on the expression of miR-148/-152 family members and DNMTs was smaller than that of metformin. However, a synergistic effect was found when they were used in combination, suggesting that cisplatin combined with metformin could modulate epigenetic changes in NSCLC.

Recent studies have demonstrated that the miR-148/-152 family may serve as a promising biomarker of prognosis in cancer patients. For example, reduction of miR-148a was independently associated with recurrence of hepatocellular carcinoma [33]. Those with low expression of miR-148a showed increased lymph node metastasis and poor clinical outcomes in NSCLC [34], bladder cancer [35], and gastric cancer [36, 37]. A meta-analysis of 24 eligible studies showed that high expression levels of miR-148a and miR-148b were associated with favorable overall survival, particularly in the Asian population [38]. Low expression level of miR-152 also was correlated with poor DFS/RFS in various cancers [39]. Inconsistent with these observations, our data showed that only miR-148b was associated with overall and recurrence-free survival in NSCLC. The lack of relationship between miR-148a and patient survival in our data might be due to the small number of patients and different criteria used for patient grouping. Further large-scale studies are needed to confirm the prognostic effect of miR-148/-152 members in NSCLC.

This study was limited by several factors. First, the molecular mechanism by which metformin regulates the expression of miR-148/-152 family members was not investigated. It has been demonstrated that miRNAs are predisposed to methylation-related silencing through a feedback loop with DNMT1, and that miRNA expression is affected by copy number alterations; another possibility is upregulation of miR-148/-152 family members through targeting of histone methyltransferases (Additional file 11). Further studies are required to demonstrate mechanisms for metformin to regulate miR-148/-152 family members in NSCLC. Second, only a subset of miR-148/-152 family members was analyzed in the transfection and luciferase assays. Third, the relationships between the expression of miR-148/-152 family members and clinicopathological variables were analyzed in a small number of samples. Further studies in a large cohort are required to confirm the results. Finally, the combined effect of cisplatin and metformin as a promising therapeutic approach to treating NSCLC needs to be tested in clinical trials.

## Conclusions

The present study suggests that metformin modulates epigenetic changes by downregulating DNMT expression through upregulation of miR-148/-152 family members in NSCLC cells. In addition, this study indicates that metformin in combination with cisplatin may synergistically regulate the expression of miR-148/-152 family members and DNMTs.

## Supplementary Information

**Additional file 1:** qPCR primer sequences

**Additional file 2:** Hierarchical clustering analysis of differentially expressed miRNAs in H1650 NSCLC cells

**Additional file 3:** Gene Ontology analysis of differentially expressed miRNAs

**Additional file 4:** Effect of miR-148b on NSCLC cell death.

**Additional file 5:** CDI of a combination of metformin and cisplatin in A549 cells

**Additional file 6:** Clinicopathological characteristics

**Additional file 7:** Correlation between age, tumor size, and the expression of miR-148/-152 family members

**Additional file 8:** Recurrence-free survival

**Additional file 9:** Cox Proportional hazards analysis

**Additional file 10:** Proposed action of metformin on cell growth

**Additional file 11:** Possible mechansim of metformin on upregulation of miR-148/-152 family members

## Abbreviations

CDI: Coefficient of drug interaction
DNMT: DNA methyltransferase
EGFR: Epidermal growth factor receptor
GO: Gene ontology
HRP: Horseradish peroxidase
KEGG: Kyoto Encyclopedia of Genes and Genomes
LUAD: Lung adenocarcinoma
mTORC1: Mammalian target of rapamycin complex 1
NSCLC: Non-small cell lung cancer
TCGA: The Cancer Genome Atlas

## References

[1] Siegel RL, Miller KD, Jemal A (2018). Cancer statistics, 2018. CA Cancer J Clin.

[2] Kim H, Kwon YM, Kim JS, Han J, Shim YM, Park J (2006). Elevated mRNA levels of DNA methyltransferase-1 as an independent prognostic factor in primary nonsmall cell lung cancer. Cancer.

[3] Lin RK, Hsu HS, Chang JW, Chen CY, Chen JT, Wang YC (2007). Alteration of DNA methyltransferases contributes to 5′CpG methylation and poor prognosis in lung cancer. Lung Cancer.

[4] Lin RK, Hsieh YS, Lin P, Hsu HS, Chen CY, Tang YA (2010). The tobacco-specific carcinogen NNK induces DNA methyltransferase 1 accumulation and tumor suppressor gene hypermethylation in mice and lung cancer patients. J Clin Invest.

[5] Liu WB, Cui ZH, Ao L, Zhou ZY, Zhou YH, Yuan XY (2011). Aberrant methylation accounts for cell adhesion-related gene silencing during 3-methylcholanthrene and diethylnitrosamine induced multistep rat lung carcinogenesis associated with overexpression of DNA methyltransferases 1 and 3a. Toxicol Appl Pharmacol.

[6] Husni RE, Shiba-Ishii A, Iiyama S, Shiozawa T, Kim Y, Nakagawa T (2016). DNMT3a expression pattern and its prognostic value in lung adenocarcinoma. Lung Cancer.

[7] Wang Y, Dai W, Chu X, Yang B, Zhao M, Sun Y (2013). Metformin inhibits lung cancer cells proliferation through repressing microRNA-222. Biotechnol Lett.

[8] Dong J, Peng H, Yang X, Wu W, Zhao Y, Chen D (2020). Metformin mediated microRNA-7 upregulation inhibits growth, migration, and invasion of non-small cell lung cancer A549 cells. Anticancer Drugs.

[9] Jin D, Guo J, Wu Y, Chen W, Du J, Yang L (2020). Metformin-repressed miR-381-YAP-snail axis activity disrupts NSCLC growth and metastasis. J Exp Clin Cancer Res.

[10] Tang G, Guo J, Zhu Y, Huang Z, Liu T, Cai J (2018). Metformin inhibits ovarian cancer via decreasing H3K27 trimethylation. Int J Oncol.

[11] Yuan H, Han Y, Wang X, Li N, Liu Q, Yin Y (2020). SETD2 restricts prostate cancer metastasis by integrating EZH2 and AMPK signaling pathways. Cancer Cell.

[12] Kim D, Kim Y, Lee BB, Cho EY, Han J, Shim TM (2021). Metformin reduces histone H3K4me3 at the promoter regions of positive cell cycle regulatory genes in lung cancer cells. Cancers (Basel).

[13] Zheng F, Wu JJ, Tang Q, Xiao Q, Wu W, Hann S (2018). The enhancement of combination of berberine and metformin in inhibition of DNMT1 gene expression through interplay of SP1 and PDPK1. J Cell Mol Med.

[14] Yan JB, Lai CC, Jhu JW, Gongol B, Marin TL, Lin SC (2020). Insulin and metformin control cell proliferation by regulating TDG-mediated DNA demethylation in Liver and breast cancer cells. Mol Ther Oncolytics.

[15] Friedrich M, Pracht K, Mashreghi MF, Jack HM, Radbruch A, Seliger B (2017). The role of the miR-148/-152 family in physiology and disease. Eur J Immunol.

[16] Braconi C, Huang N, Patel T (2010). MicroRNA-dependent regulation of DNA methyltransferase-1 and tumor suppressor gene expression by interleukin-6 in human malignant cholangiocytes. Hepatology.

[17] Wang X-X, Zhang H, Li Y (2019). Preliminary study on the role of miR-148a and DNMT1 in the pathogenesis of acute myeloid leukemia. Mol Med Rep.

[18] Zhu A, Xia J, Zuo J, Jin S, Zhou H, Yao L (2012). MicroRNA-148a is silenced by hypermethylation and interacts with DNA methyltransferase 1 in gastric cancer. Med Oncol.

[19] Long XR, He Y, Huang C, Li J (2014). MicroRNA-148a is silenced by hypermethylation and interacts with DNA methyltransferase 1 in hepatocellular carcinogenesis. Int J Oncol.

[20] Hong L, Sun G, Peng L, Tu Y, Wan Z, Xiong H (2018). The interaction between miR-148a and DNMT1 suppresses cell migration and invasion by reactivating tumor suppressor genes in pancreatic cancer. Oncol Rep.

[21] Sengupta D, Deb M, Patra SK (2018). Antagonistic activities of miR-148a and DNMT1: Ectopic expression of miR-148a impairs DNMT1 mRNA and dwindle cell proliferation and survival. Gene.

[22] Wang X, Liang Z, Xu X, Li J, Zhu Y, Meng S (2016). miR-148a-3p represses proliferation and EMT by establishing regulatory circuits between ERBB3/AKT2/c-myc and DNMT1 in bladder cancer. Cell Death Dis.

[23] Chen Q, Wang Y, Dang H, Wu X (2021). MicroRNA-148a-3p inhibits the proliferation of cervical cancer cells by regulating the expression levels of DNMT1 and UTF1. Oncol Lett.

[24] Azizi M, Teimoori-Toolabi L, Arzanani MK, Azadmanesh K, Fard-Esfahani P, Zeinali S (2014). MicroRNA-148b and microRNA-152 reactivate tumor suppressor genes through suppression of DNA methyltransferase-1 gene in pancreatic cancer cell lines. Cancer Biol Ther.

[25] Sui C, Meng F, Li Y, Jiang Y (2015). miR-148b reverses cisplatin-resistance in non-small cell cancer cells via negatively regulating DNA (cytosine-5)-methyltransferase 1(DNMT1) expression. J Transl Med.

[26] Mou Z, Xu X, Dong M, Xu J (2016). MicroRNA-148b acts as a tumor suppressor in cervical cancer by inducing G1/S-phase cell cycle arrest and apoptosis in a caspase-3-dependent manner. Med Sci Monit.

[27] Chen X, Ma X, Zhang L (2020). MicorRNA-148b inhibits cell proliferation and facilitates cell apoptosis by regulating DNA Methyltransferase 1 in endometrial cancer. Transl Cancer Res.

[28] Huang J, Wang Y, Guo Y, Sun S (2010). Down-regulated microRNA-152 induces aberrant DNA methylation in hepatitis B virus-related hepatocellular carcinoma by targeting DNA methyltransferase 1. Hepatology.

[29] Xiang Y, Ma N, Wang D, Zhang Y, Zhou J, Wu G (2014). MiR-152 and miR-185 co-contribute to ovarian cancer cells cisplatin sensitivity by targeting DNMT1 directly; a novel epigenetic therapy independent of decitabine. Oncogene.

[30] Sun J, Tian X, Zhang J, Huang Y, Lin X, Chen L (2017). Regulation of human glioma cell apoptosis and invasion by miR-152-3p through targeting DNMT1 and regulating NF2. J Exp Clin Cancer Res.

[31] Lu ZW, Du MY, Qian LX, Zhang N, Gu JJ, Ding K (2018). MiR-152 functioning as a tumor suppressor that interacts with DNMT1 in nasopharyngeal carcinoma. Onco Targets Ther.

[32] Zhang H, Qi D, Li J, Peng T, Yang L, Yuan J (2018). A novel regulatory circuit of miR-152 and DNMT1 in human bladder cancer. Oncol Rep.

[33] Ajdarkosh H, Dadpay M, Yahaghi E, Pirzaman ER, Fayyaz AF, Darian EK (2015). Decreased expression and clinicopathological significance of miR-148a with poor survival in hepatocellular carcinoma tissues. Diagn Pathol.

[34] Chen Y, Min L, Zhang X, Hu S, Wang B, Liu W (2013). Decreased miRNA-148a is associated with lymph node metastasis and poor clinical outcomes and functions as a suppressor of tumor metastasis in non-small cell lung cancer. Oncol Rep.

[35] Ma L, Xu Z, Xu C, Jiang X (2016). Micro-RNA148a represents an independent prognostic marker in bladder cancer. Tumour Biol.

[36] Qiu X, Zhu H, Liu S, Tao G, Jin J, Chu H (2017). Expression and prognostic value of microRNA-26a and microRNA-148a in gastric cancer. J Gastroenterol Hepatol.

[37] Komatsu S, Imamura T, Kiuchi J, Takashima Y, Kamiya H, Ohashi T (2021). Depletion of tumor suppressor miRNA-148a in plasma relates to tumor progression and poor outcomes in gastric cancer. Am J Cancer Res.

[38] Miao C, Zhang J, Zhao K, Liang C, Xu A, Zhu J (2017). The significance of microRNA-148/152 family as a prognostic factor in multiple human malignancies: a meta analysis. Oncotarget.

[39] Duan F, Liu W, Fu X, Feng Y, Dai L, Cui S (2017). Evaluating the prognostic value of miR-148/152 family in cancers: based on a systemic review of observational studies. Oncotarget.

