# Supplementary material for: Metformin regulates expression of DNA methyltransferases through the miR-148/-152 family in non-small lung cancer cells
Source: Clin Epigenetics. 2023 Mar 23;15:48. doi: 10.1186/s13148-023-01466-0 (PMC10037810; doi:10.1186/s13148-023-01466-0)
Supplement: Supplementary file 1 — Additional file 1: qPCR primer sequences [file 13148_2023_1466_MOESM1_ESM.docx]

**Additional file 1. qPCR primer sequences**

|  | Forward | Reverse |
| --- | --- | --- |
| GAPDH | GAAGGTGAAGGTCGGAGTC | GAAGATGGTGATGGGATTTC |
| DNMT1 | TACCTGGACGACCCTGACCTC | CGTTGGCATCAAAGATGGACA |
| DNMT3a | TATTGATGAGCGCACAAGAGAGC | GGGTGTTCCAGGGTAACATTGAG |
| DNMT3b | TATTGATGAGCGCACAAGAGAGC | GGCAAGTTCTCCGAGGTCTCTG |
| U6 | GCTTCGGCAGCACATATACTAAAAT | CGCTTCACGAATTTGCGTGTCAT |
| miR-148a | MIMAT0000243: 5'UCAGUGCACUACAGAACUUUGU | |
| miR-148b | MIMAT0000759: 5'UCAGUGCAUCACAGAACUUUGU | |
| miR-152 | MIMAT0000438: 5'UCAGUGCAUGACAGAACUUGG | |

miRNA primers were purchased from QIAGEN.
