# Supplementary figures and images for: Metformin regulates expression of DNA methyltransferases through the miR-148/-152 family in non-small lung cancer cells
Source: Clin Epigenetics. 2023 Mar 23;15:48. doi: 10.1186/s13148-023-01466-0 (PMC10037810; doi:10.1186/s13148-023-01466-0)

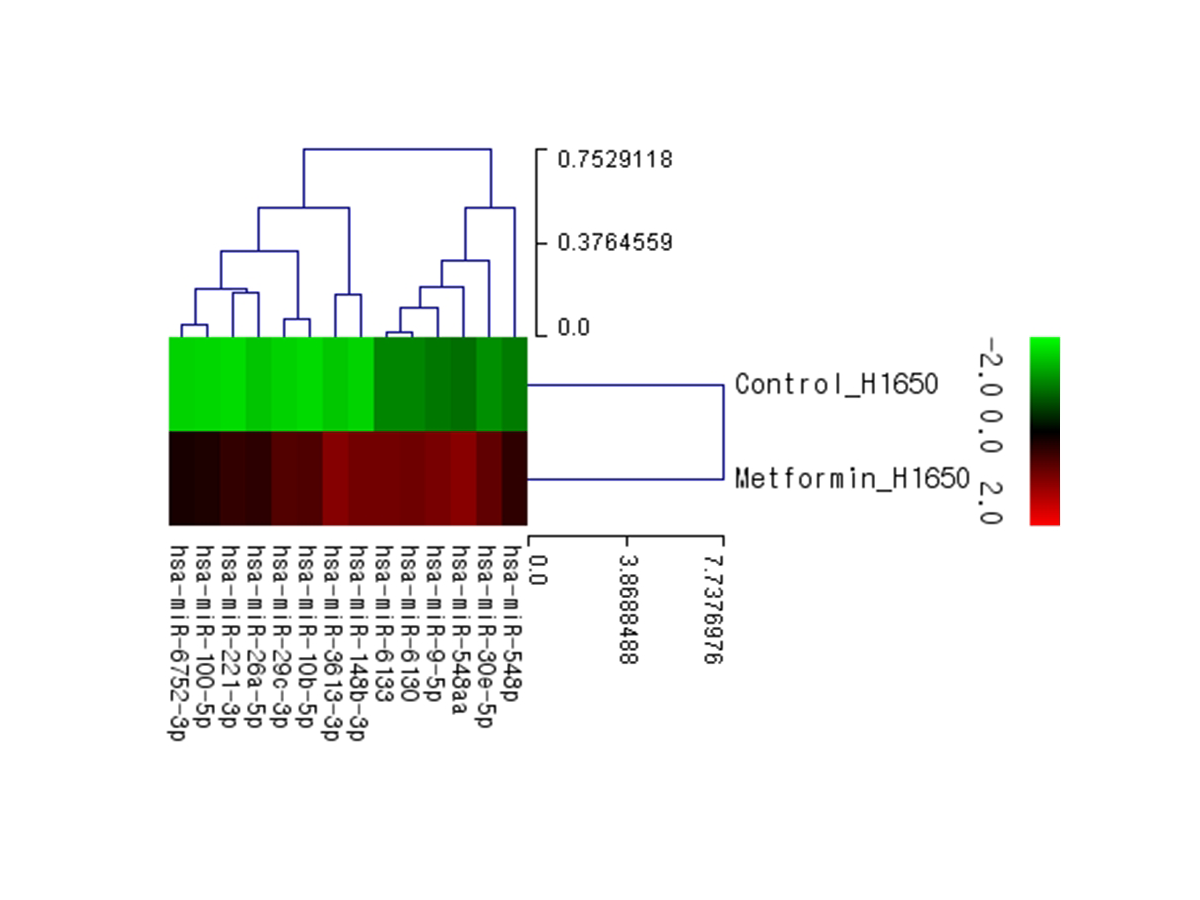

Supplement: Supplementary file 2 — Additional file 2: Hierarchical clustering analysis of differentially expressed miRNAs in H1650 NSCLC cells [file 13148_2023_1466_MOESM2_ESM.tif]

**Additional file 3. Gene Ontology analysis of differentially expressed miRNAs**

(1) A549 cells


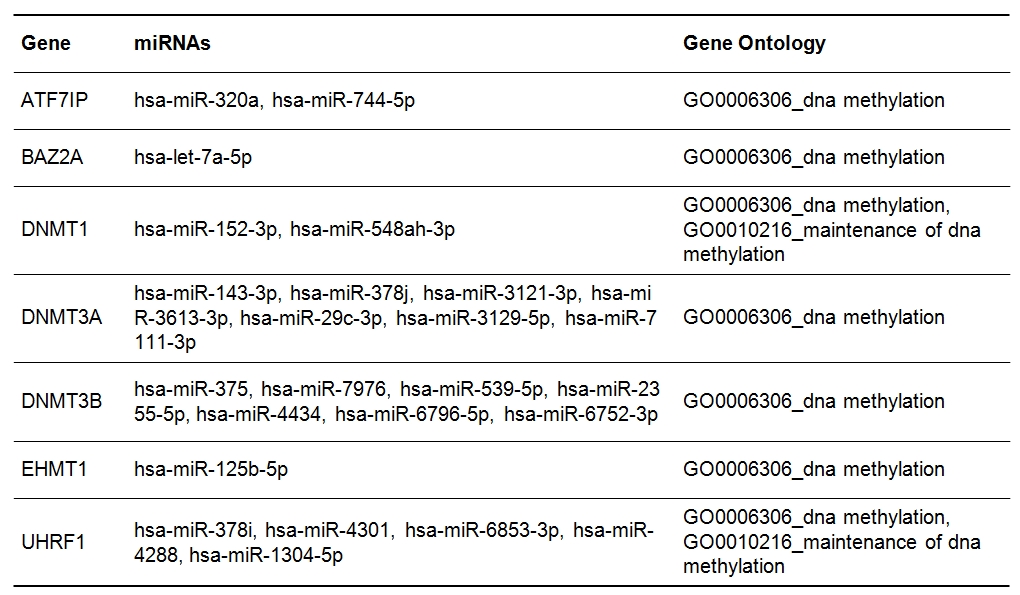


(2) H1650 cells


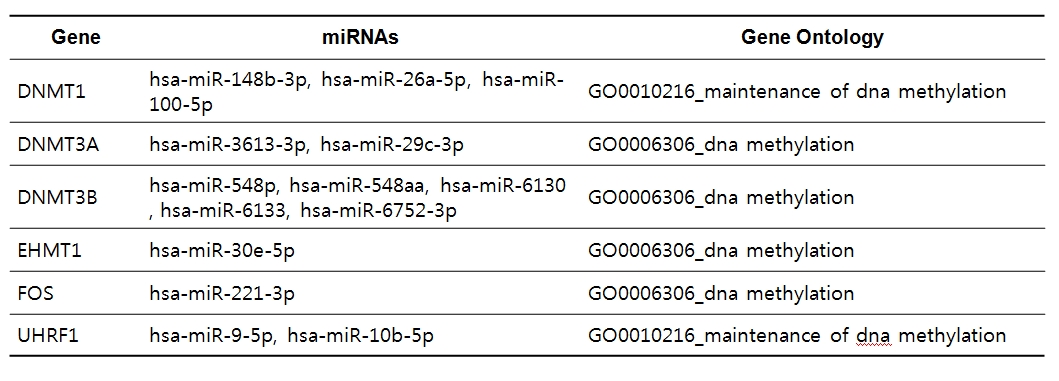

Supplement: Supplementary file 3 — Additional file 3: Gene Ontology analysis of differentially expressed miRNAs [file 13148_2023_1466_MOESM3_ESM.docx]

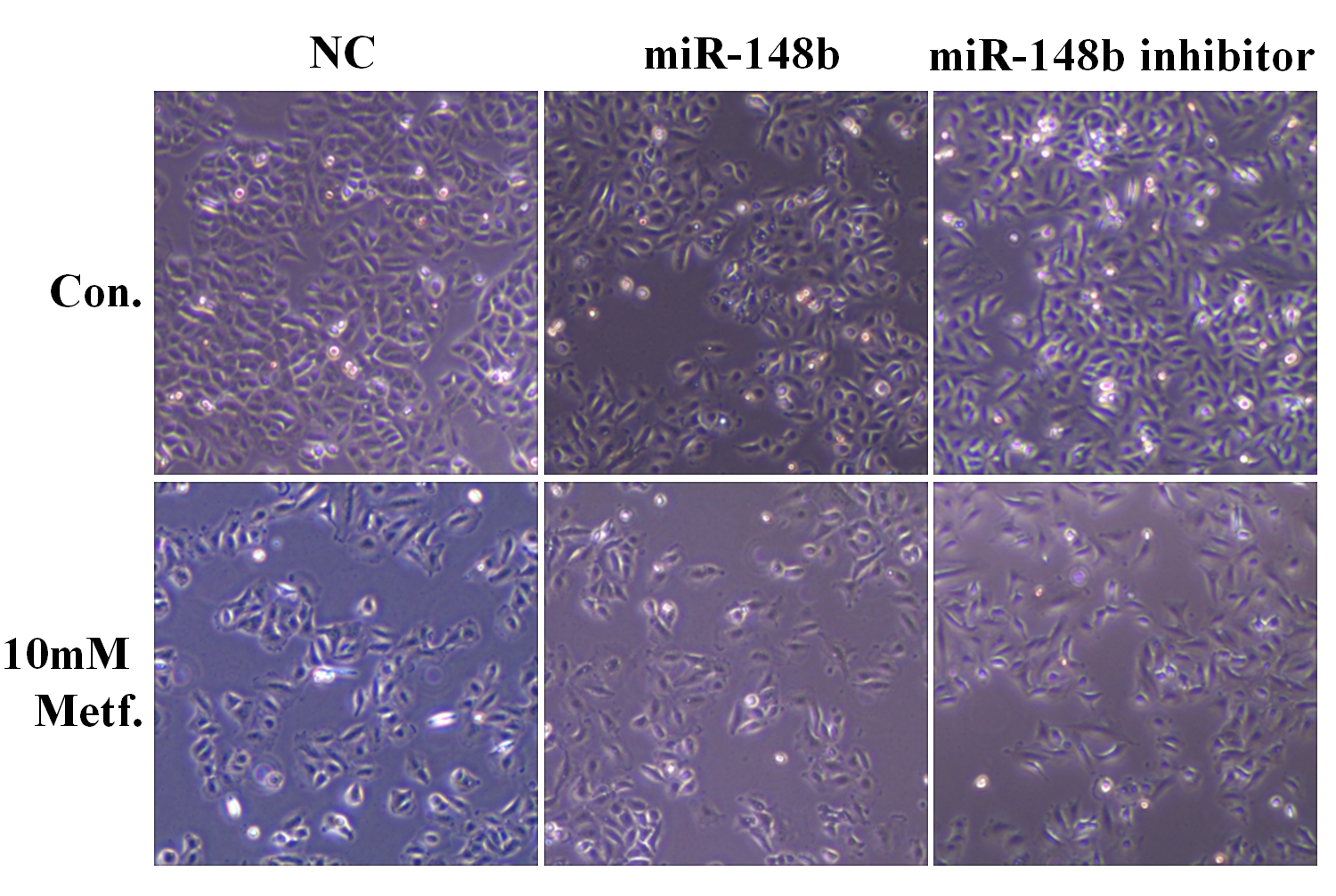

Supplement: Supplementary file 4 — Additional file 4: Effect of miR-148b on NSCLC cell death. [file 13148_2023_1466_MOESM4_ESM.tif]

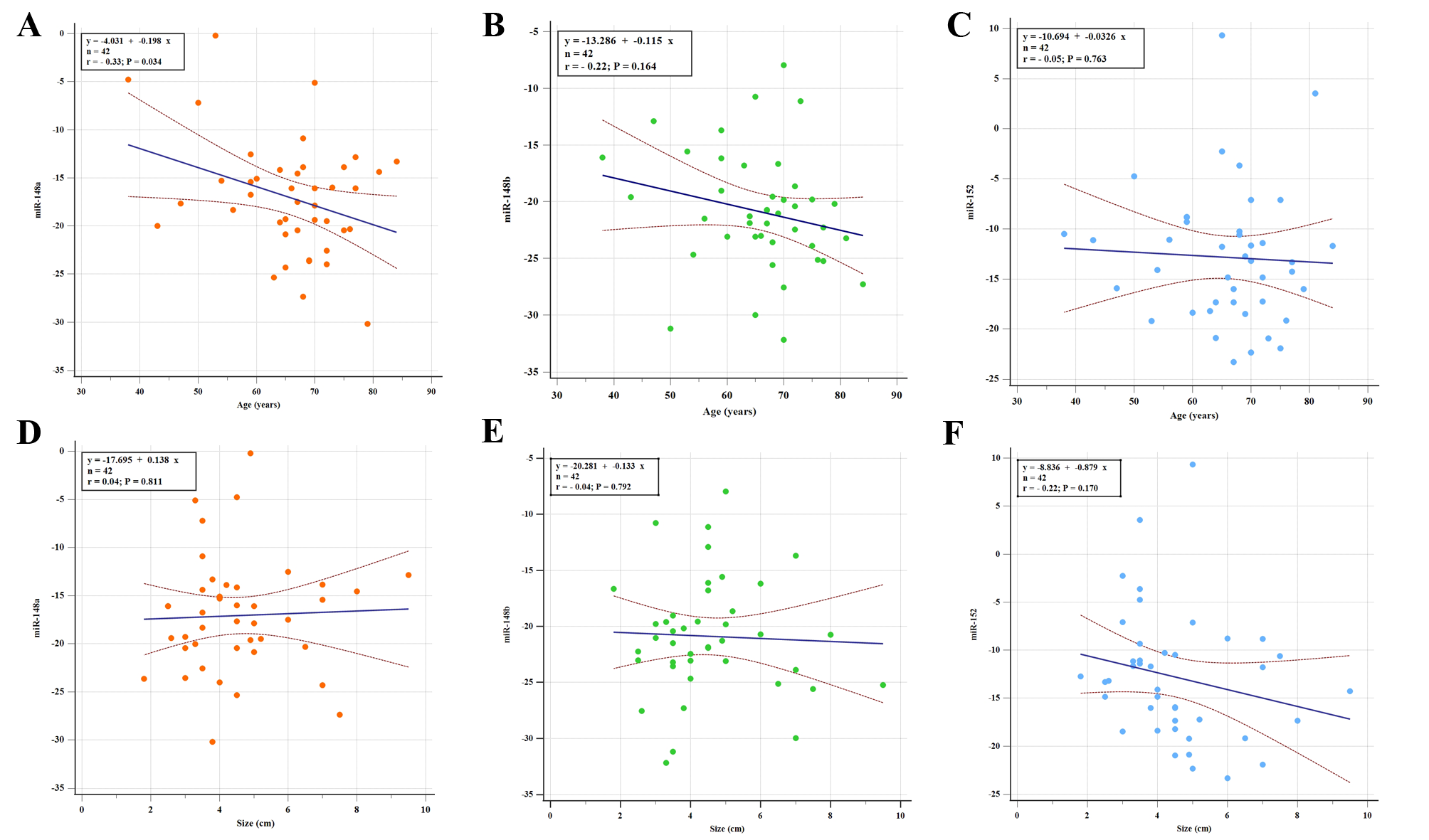

Supplement: Supplementary file 7 — Additional file 7: Correlation between age, tumor size, and the expression of miR-148/-152 family members [file 13148_2023_1466_MOESM7_ESM.tif]

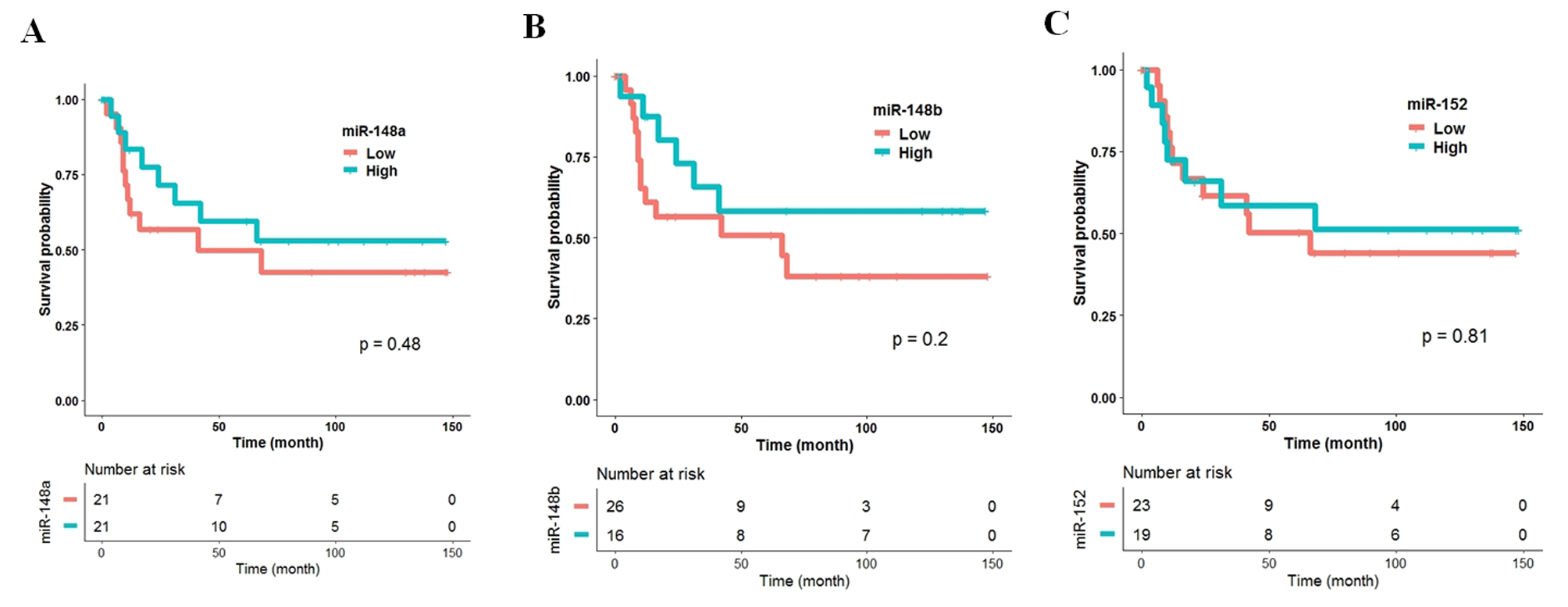

Supplement: Supplementary file 8 — Additional file 8: Recurrence-free survival [file 13148_2023_1466_MOESM8_ESM.tif]

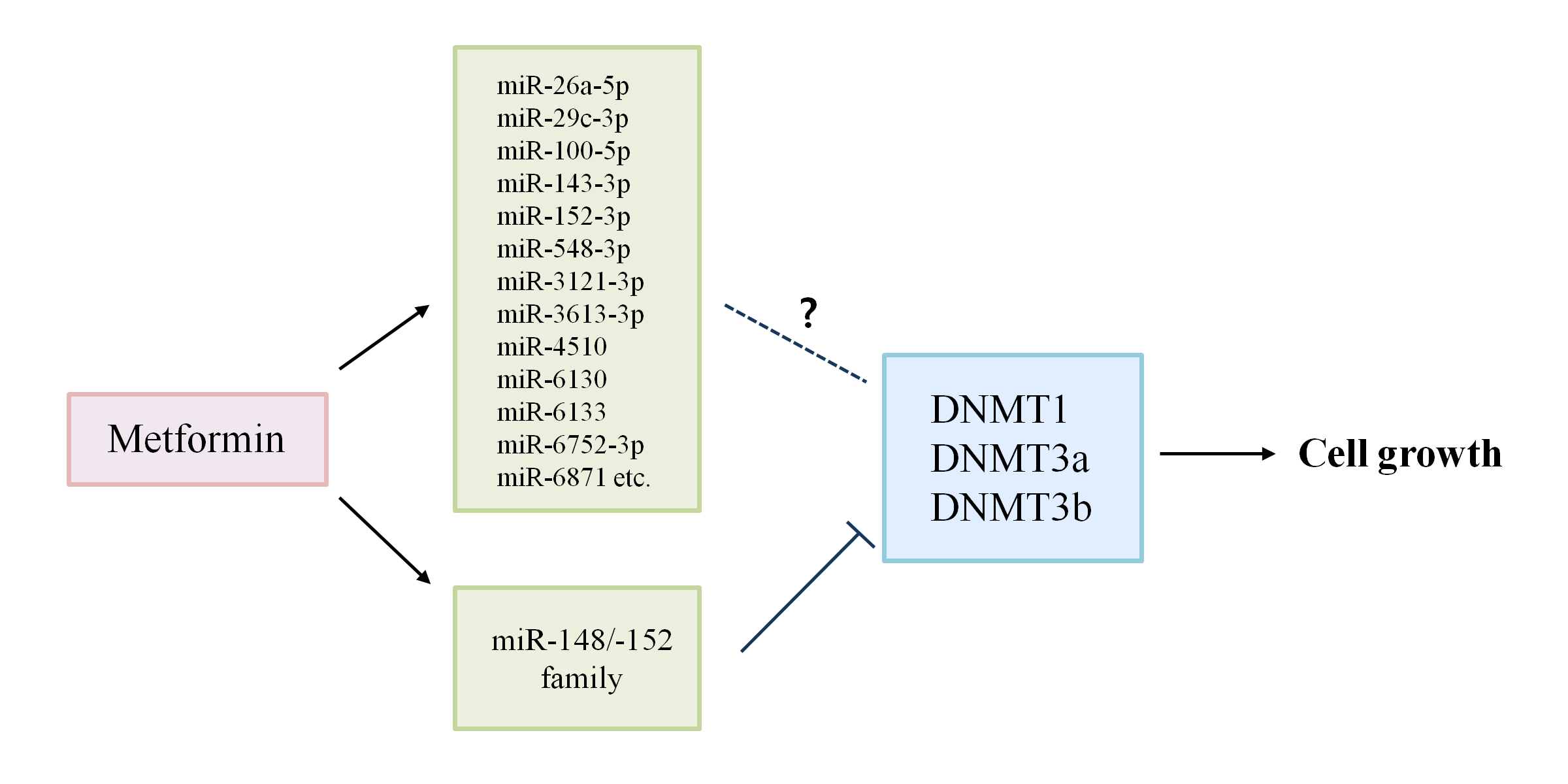

Supplement: Supplementary file 10 — Additional file 10: Proposed action of metformin on cell growth [file 13148_2023_1466_MOESM10_ESM.tif]

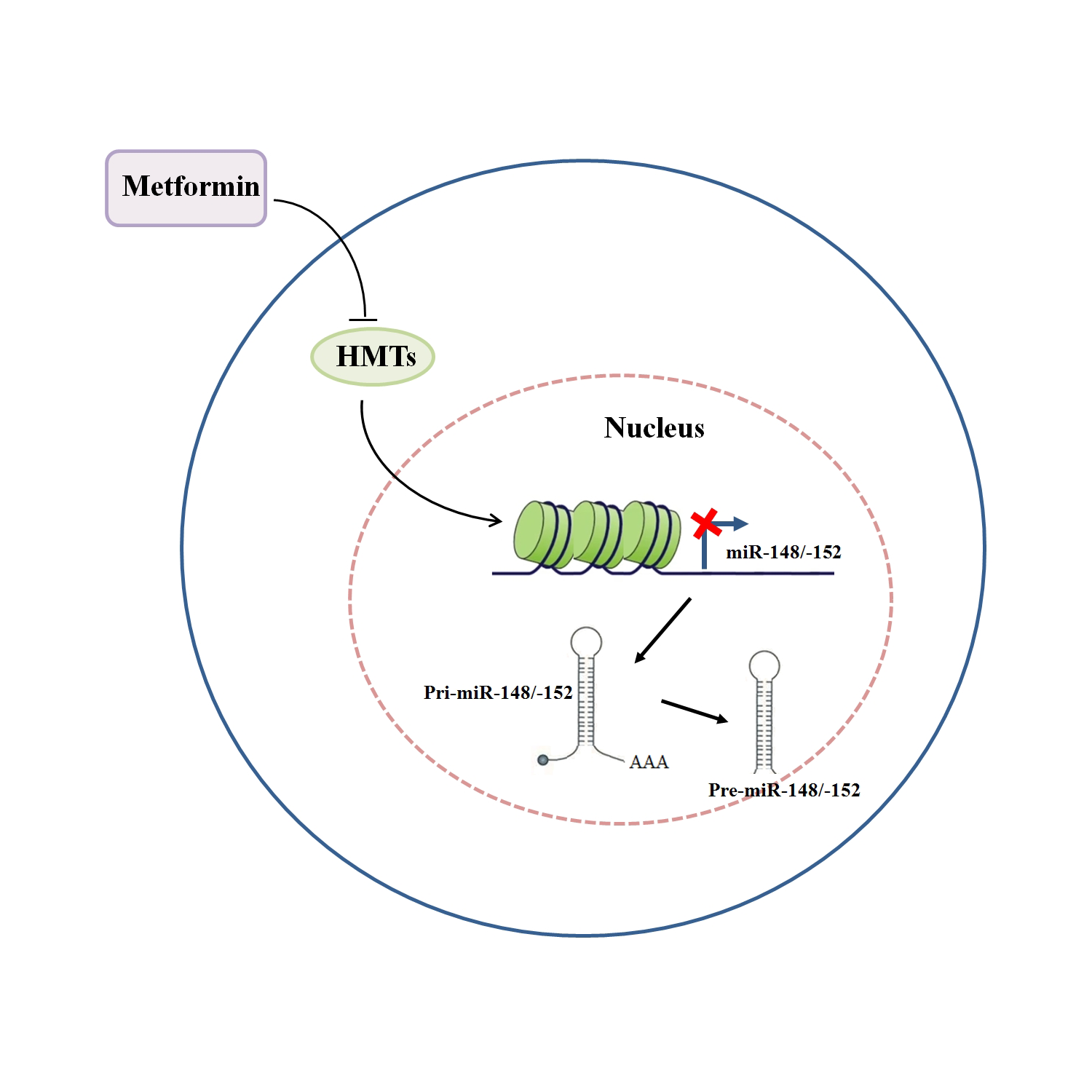

Supplement: Supplementary file 11 — Additional file 11: Possible mechansim of metformin on upregulation of miR-148/-152 family members [file 13148_2023_1466_MOESM11_ESM.tif]
