# Supplementary material for: Metformin regulates expression of DNA methyltransferases through the miR-148/-152 family in non-small lung cancer cells
Source: Clin Epigenetics. 2023 Mar 23;15:48. doi: 10.1186/s13148-023-01466-0 (PMC10037810; doi:10.1186/s13148-023-01466-0)
Supplement: Supplementary file 5 — Additional file 5: CDI of a combination of metformin and cisplatin in A549 cells [file 13148_2023_1466_MOESM5_ESM.docx]

**Additional file 5. CDI of a combination of metformin and cisplatin in A549 cells**

|  | | | |  |  |
| --- | --- | --- | --- | --- | --- |
| **Metformin**  **(mM)** | **Cisplatin**  **(uM)** | **Growth inhibitory effects (OD)** | |  | **CDI** |
|  |  | **Metformin** | **Cisplatin** | **Metformin**  **+ Cisplatin.** |  |
| 0 | 0 | 1.59 ± 0.20 | 1.59 ± 0.20 | 1.59 ± 0.20 |  |
| 5 | 6.25 | 1.13 ± 0.13 | 1.80 ± 0.07 | 1.27 ± 0.06 | 0.62 |
| 5 | 12.5 |  | 1.72 ± 0.07 | 1.07 ± 0.09 | 0.55 |
| 5 | 25 |  | 1.62 ± 0.14 | 0.75 ± 0.06 | 0.41 |
| 5 | 50 |  | 1.25 ± 0.10 | 0.58 ± 0.11 | 0.41 |
| 10 | 6.25 | 1.06 ± 0.07 | 1.80 ± 0.07 | 1.24 ± 0.12 | 0.65 |
| 10 | 12.5 |  | 1.72 ± 0.07 | 1.07 ± 0.07 | 0.58 |
| 10 | 25 |  | 1.62 ± 0.14 | 0.48 ± 0.13 | 0.28 |
| 10 | 50 |  | 1.25 ± 0.10 | 0.60 ± 0.05 | 0.45 |
| 20 | 6.25 | 1.26 ± 0.12 | 1.80 ± 0.07 | 1.32 ± 0.09 | 0.58 |
| 20 | 12.5 |  | 1.72 ± 0.07 | 0.95 ± 0.14 | 0.44 |
| 20 | 25 |  | 1.62 ± 0.14 | 0.75 ± 0.08 | 0.37 |
| 20 | 50 |  | 1.25 ± 0.10 | 0.28 ± 0.06 | 0.18 |
