# Supplementary material for: Metformin regulates expression of DNA methyltransferases through the miR-148/-152 family in non-small lung cancer cells
Source: Clin Epigenetics. 2023 Mar 23;15:48. doi: 10.1186/s13148-023-01466-0 (PMC10037810; doi:10.1186/s13148-023-01466-0)
Supplement: Supplementary file 6 — Additional file 6: Clinicopathological characteristics [file 13148_2023_1466_MOESM6_ESM.docx]

**Additional file 6. Clinicopathological characteristics of 42 NSCLC patients**

| **Variables** | **N** | **miR-148a** | ***P*** | **miR-148b** | ***P*** | **miR-152** | ***P*** |
| --- | --- | --- | --- | --- | --- | --- | --- |
| Sex |  |  |  |  |  |  |  |
| Men | 27 | -17.9 ± 4.8 |  | -21.1 ± 4.9 |  | -12.8 ± 7.6 |  |
| Women | 15 | -15.6 ± 7.6 | 0.31 | -20.4 ± 5.9 | 0.69 | -12.8 ± 4.9 | 0.99 |
| pStage |  |  |  |  |  |  |  |
| I | 26 | -18.1 ± 5.1 |  | -20.3 ± 5.3 |  | -12.5 ± 5.9 |  |
| II | 15 | -15.5 ± 7.3 |  | -22.0 ± 5.2 |  | -13.1 ± 8.3 |  |
| III | 1 | -14.5 ± 0.0 | 0.39 | -20.8 ± 0.0 | 0.62 | -17.3 ± 0.0 | 0.77 |
| IV |  |  |  |  |  |  |  |
| Status |  |  |  |  |  |  |  |
| Never | 14 | -15.1 ± 7.6 |  | -21.6 ± 6.9 |  | -13.0 ± 5.1 |  |
| Former | 16 | -18.1 ± 5.1 |  | -19.5 ± 4.9 |  | -11.2 ± 7.6 |  |
| Current | 12 | -17.9 ± 4.7 | 0.34 | -21.9 ± 3.5 | 0.42 | -14.7 ± 7.2 | 0.40 |
| Hist |  |  |  |  |  |  |  |
| Adeno | 27 | -16.6 ± 5.8 |  | -20.9 ± 6.2 |  | -11.4± 5.6 |  |
| Squamous | 8 | -18.4 ± 4.9 |  | -21.1 ± 3.4 |  | -16.5 ± 4.2 |  |
| Others | 7 | -17.2 ± 8.1 | 0.76 | -20.6 ± 2.8 | 0.98 | -14.3 ± 10.9 | 0.14 |
| L_inv |  |  |  |  |  |  |  |
| No | 19 | -18.5 ± 4.7 |  | -20.8 ± 6.1 |  | -14.7 ± 5.0 |  |
| Yes | 23 | -15.9 ± 6.8 | 0.17 | -20.9 ± 4.5 | 0.96 | -11.3 ± 7.6 | 0.11 |
| LN meta |  |  |  |  |  |  |  |
| No | 35 | -17.8 ± 5.4 |  | -20.9 ± 5.2 |  | -12.7 ± 6.9 |  |
| Yes | 7 | -14.9 ± 6.9 | 0.31 | -20.6 ± 5.5 | 0.87 | -13.8 ± 5.9 | 0.70 |
| V_inv |  |  |  |  |  |  |  |
| No | 34 | -17.7 ± 5.8 |  | -21.4 ± 5.5 |  | -12.8 ± 7.2 |  |
| Yes | 8 | -14.5 ± 6.4 | 0.18 | -18.8 ± 3.3 | 0.21 | -13.1 ± 4.7 | 0.89 |
| N_inv |  |  |  |  |  |  |  |
| No | 38 | -17.4 ± 5.8 |  | -21.3 ± 5.2 |  | -13.1 ± 7.0 |  |
| Yes | 4 | -14.2 ± 7.9 | 0.32 | -17.1 ± 3.7 | 0.13 | -10.7 ± 2.8 | 0.52 |

Abbreviations: Adeno, adenocarcinoma; Squamous, squamous cell carcinoma; L_inv, lymphatic invasion; LN meta, lymph node metastasis; V-inv, venous invasion; N-inv, Nervous invasion.

Data are shown as mean ± standard deviation

*P*-values are based on t-test or ANOVA.
