# Supplementary material for: Metformin regulates expression of DNA methyltransferases through the miR-148/-152 family in non-small lung cancer cells
Source: Clin Epigenetics. 2023 Mar 23;15:48. doi: 10.1186/s13148-023-01466-0 (PMC10037810; doi:10.1186/s13148-023-01466-0)
Supplement: Supplementary file 9 — Additional file 9: Cox Proportional hazards analysis [file 13148_2023_1466_MOESM9_ESM.docx]

**Additional file 9. Cox Proportional hazards analysis**

**(A) Overall survival**

| miRNAs | Expression | HR^a^ | 95% CI | *P*-value |
| --- | --- | --- | --- | --- |
| miR-148a | high | 1.00 |  |  |
|  | low | 1.21 | 0.52 - 2.46 | 0.81 |
| miR-148b | high | 1.00 |  |  |
|  | low | 2.56 | 1.09 - 6.47 | 0.04 |
| miR-152 | high | 1.00 |  |  |
|  | low | 1.28 | 0.58 - 2.89 | 0.56 |

Abbreviations: HR, hazard ratio; CI, confidence interval

^a^adjusted for age, sex, pathologic stage, smoking status, histology, and

recurrence

**(B) Recurrence-free survival**

| miRNAs | Expression | HR^a^ | 95% CI | *P*-value |
| --- | --- | --- | --- | --- |
| miR-148a | high | 1.00 |  |  |
|  | low | 1.63 | 0.61 - 4.37 | 0.33 |
| miR-148b | high | 1.00 |  |  |
|  | low | 1.24 | 0.44 - 3.51 | 0.68 |
| miR-152 | high | 1.00 |  |  |
|  | low | 1.12 | 0.45 - 2.78 | 0.29 |

Abbreviations: HR, hazard ratio; CI, confidence interval

^a^adjusted for age, sex, pathologic stage, and histology
